# Fissures of the Anus in Children

**Published:** 1892-02

**Authors:** 


### FISSURES OF THE ANUS IN CHILDREN.

Dr. Braun (*Hospitals-Tidende*, R. 3, Bd. 8, S. 1889) finds the use of lime-water as an addition to milk a frequent cause of constipation, and consequent fissures of the anus in children. Any cause which produces diarrhœa with following constipation will cause it. Constipation, proctitis and severe pains on defecation are the results of a fissure. Hernia and masturbation are possible consequences. The condition may be long lasting, although it is easily discovered when attention is called to it He treats it by regulating the diet, cleanliness, irrigation of the rectum, and dilatation of the sphincter ani, which is easily done.—*Ex.*

